# Plasma proteomic and metabolomic signatures of B‐ALL patients during CAR‐T cell therapy

**DOI:** 10.1002/ctm2.1225

**Published:** 2023-03-20

**Authors:** Jianghua Wu, Lu Tang, Mengyi Du, Chenggong Li, Haiming Kou, Huiwen Jiang, Wenjing Luo, Yinqiang Zhang, Zhongpei Huang, Danying Liao, Wei Xiong, Heng Mei, Yu Hu

**Affiliations:** ^1^ Institute of Hematology Union Hospital Tongji Medical College Huazhong University of Science and Technology Wuhan China; ^2^ Hubei Clinical Medical Center of Cell Therapy for Neoplastic Disease Wuhan China

Dear Editor,

Immunity‐related adverse events and prognostic heterogeneity remain key obstacles to chimeric antigen receptor (CAR)‐T cell therapy. These issues have been investigated at cellular, genomic and transcriptomic levels.^1^, ^2^, ^3^ But, studies regarding plasma proteomic and metabolomic changes during CAR‐T cell therapy are scanty. This study first revealed the landscape of plasma proteome and metabolome in patients with B‐cell acute lymphoblastic leukemia (B‐ALL) during CAR‐T cell therapy and further provided molecular annotations for CAR‐T‐related adverse events and prognostic heterogeneity.

In this study, we longitudinally profiled the plasma proteome and metabolome of 20 B‐ALL patients ‐receiving CAR‐T cell therapy. CAR structure was mainly composed of humanized CD19 single‐chain variable fragment, CD3ζ transmembrane domain and 4‐1BB costimulatory domain. The entire workflow is shown in Supplemental Figure S1. More information is detailed in the Supporting Information. The clinical characteristics of B‐ALL patients and healthy controls (HC) are detailed in Supplemental Table S1. After receiving CAR‐T cells, 19 (95.0%) patients achieved complete remission (CR) within 1 month (Supplemental Figure S2A). During subsequent follow‐up, a total of five patients died of disease progression. Of them, one patient did not respond to CAR‐T cell infusion and four died of post‐treatment relapses (including two positive relapses and two negative relapses). All patients experienced cytopenia over the course of CAR‐T cell therapy (Supplemental Table S2; Supplemental Figure S2B). Two (10.0%) patients suffered from CAR‐T‐cell‐related encephalopathy syndrome (CRES); 13 (65.0%) patients developed cytokine release syndrome (Supplemental Table S2). The greatest expansion of CAR‐T cells was found at day 14 post‐infusion (Supplemental Figure S2C). The IL‐6 level correlated with high tumour burden (Supplemental Figure S2D‐E).

We first investigated the plasma proteomic signature of B‐ALL patients relative to HC. There were 239, 217, 282, 260 and 261 differentially expressed proteins (DEPs) in these five groups, respectively (Figure 1A). Most of DEPs were downregulated in B‐ALL patients and highly enriched in actin binding and actin cytoskeleton organization, among others (Figure 1B,C; Supplemental Figure S3). The upregulated proteins in B‐ALL patients predominated in acute phase response, with more upregulated proteins at day 7 post‐infusion (Supplemental Figures S3 and S4; Supplemental Table S3). The proteins, which were involved in regulation of lipid localization, were upregulated at day 14 post‐infusion, including apolipoprotein C4, C‐reactive protein and secreted phosphoprotein 1 (Supplemental Figure S3E; Supplemental Table S4). In our metabolomic profiling, 104, 139, 175, 223 and 87 differentially expressed metabolites (DEMs) were found in these five groups, respectively (Figure 1D). The enriched pathways of DEMs varied at the different time points (Figure 1E; Supplemental Table S5). For instance, DEMs were highly enriched in glycosylphosphatidylinositol‐anchor biosynthesis at day 7 post‐infusion (Figure 1E). The downregulated lipids were mainly glycerophospholipids, carnitines and sphingolipids, while the upregulated metabolites included diglycerides (DGs), triglycerides (TGs) and purine metabolites in B‐ALL patients (Figure 1F; Supplemental Figure S5; Supplemental Table S6). Noteworthily, both DEPs and DEMs were enriched in autophagy and downregulated in B‐ALL patients (Figure 1B,E; Supplemental Figures S5 and S6).

**FIGURE 1:**
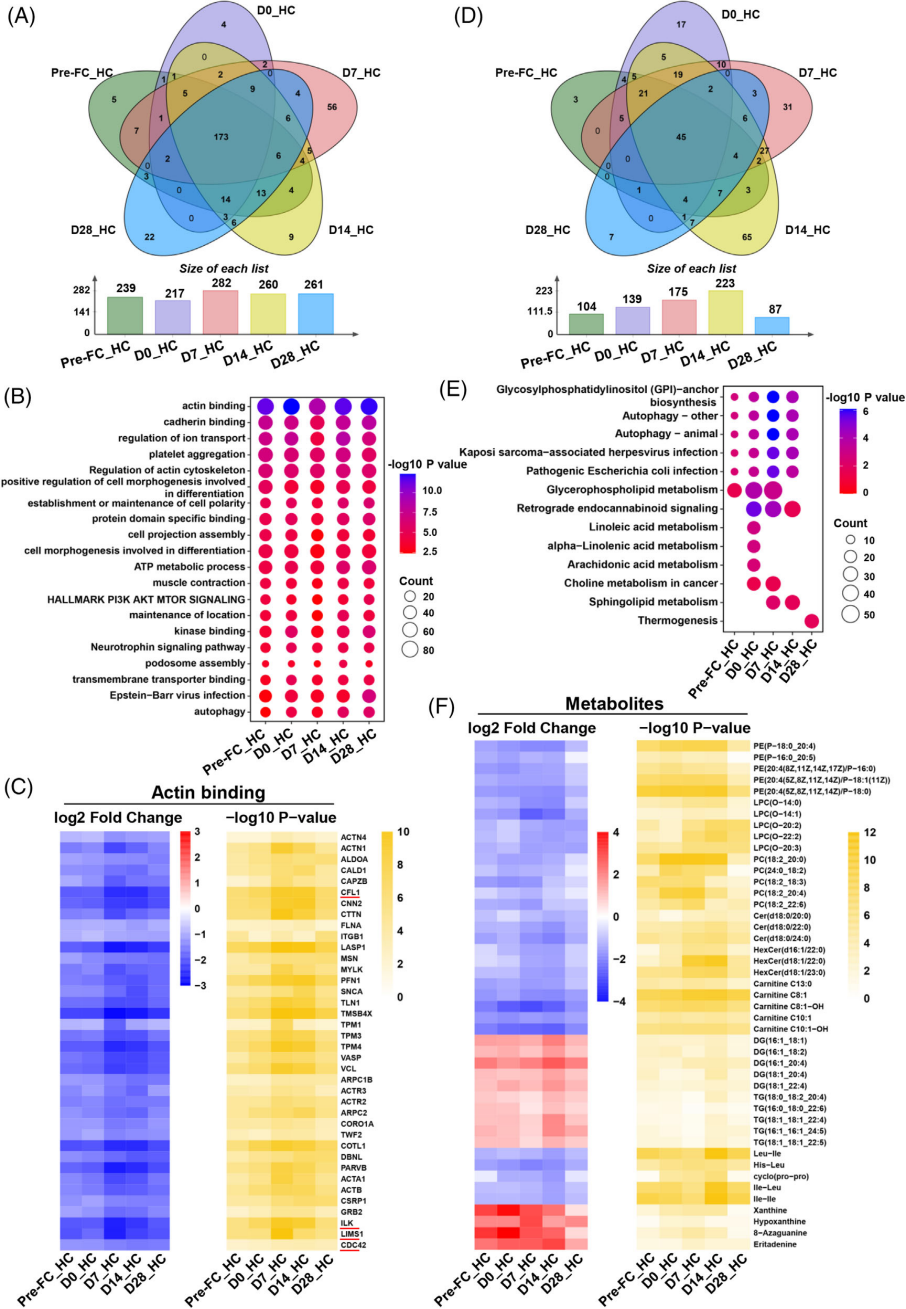
Plasma proteomic and metabolomic profiles differ between B‐ALL patients and healthy controls. (A) The Venn diagram and the histogram display the number of DEPs in the comparisons of pre‐FC versus HC, D0 versus HC, D7 versus HC, D14 versus HC and D28 versus HC. The criteria for DEPs selection were that the *p* value should be less than 0.05 and |log2 fold change| should be larger than 0.585. (B) Pathway enrichment analyses of DEPs in term of biological processes are presented in the bubble chart. (C) The heatmap shows the values of the log of the fold change of selected plasma proteins involved in actin binding and corresponding *p* values for the B‐ALL patients versus HC at different time points. (D) The Venn diagram and the histogram show the number of DEMs in the comparisons of pre‐FC versus HC, D0 versus HC, D7 versus HC, D14 versus HC and D28 versus HC groups. The DEMs were determined by VIP ≥ 1, *p* value < 0.05 and |log2 (fold change)| ≥ 1. (E) KEGG‐based enrichment analysis of DEMs is presented in the bubble chart. (F) The heatmap exhibits the values of the log of the fold change of selected plasma metabolites and corresponding *p* values for the B‐ALL patients versus HC at different time points. HC means healthy controls. Pre‐FC represents the time point when the samples were collected before the fludarabine/cyclophosphamide lymphodepletion. D0, D7, D14 and D28 represent the time points when the samples were collected on the 0, 7th, 14th and 28th day after CAR‐T cell infusion.

Given the significant differences in plasma molecular signatures between B‐ALL patients and HC, we next performed plasma proteomic and metabolomic analyses of samples collected from the different time points relative to pretreatment samples. Compared with pretreatment samples, most of DEPs were decreased in B‐ALL patients and enriched in regulation of cytoskeleton organization at day 7 and 14 post‐infusion (Supplemental Figure S7A‐7B). As expected, the upregulated DEPs were observed in B‐ALL patients at day 7 post‐infusion; these upregulated proteins were enriched in pathways including regulation of the immune effector process, collagen metabolic process and hallmark peroxisome (Supplemental Figure S7A‐7B, Supplemental Table S7). In our metabolomic profiling, the pathway enrichment of DEMs revealed that histidine metabolism, biosynthesis of cofactors, 2‐oxocarboxylic acid metabolism and biosynthesis of amino acid were disturbed at day 7 post‐infusion, while purine metabolism was dysregulated at day 14 post‐infusion (Supplemental Figure S7C‐7D).

To elucidate the dynamics of plasma proteins and metabolites during CAR‐T cell therapy, we conducted co‐expression clustering analyses (Figure 2A–D; Supplemental Tables S8 and S9). Whole proteome cluster 1 (WPC1) showed that the proteins, which were enriched in positive regulation of heterotypic cell–cell adhesion, were gradually decreased after CAR‐T cell infusion (Figure 2C,E). Axon extension, intramolecular oxidoreductase activity and regulation of GTPase activity were observed in WPC2 (Figure 2A,C), suggesting that these pathways were suppressed during CAR‐T cell therapy. Actin cytoskeleton organization was identified in WPC3 (Figure 2A,C,E), suggesting that cytoskeleton organization was disrupted. WPC4 contained the acute‐phase response that represents a quick response of the body to CAR‐T cell infusion (Figure 2C,E). In our metabolomic profiling, TGs were found in whole metabolome cluster 1 (WMC1) and highly enriched in lipid and atherosclerosis, regulation of lipolysis in adipocytes and cholesterol metabolism (Figure 2D; Supplemental Tables S9 and S10). An elevated plasma TGs level may result from adipose tissue lipolysis under hyperinflammatory condition.^4^ Free fatty acids and purine metabolites were identified in whole metabolome cluster 2 (WMC2) (Figure 2B; Supplemental Table S10). Phosphatidylcholines (PC) and phosphatidylethanolamines (PE) were observed in whole metabolome cluster 3 (WMC3) and highly enriched in retrograde endocannabinoid signalling and autophagy, among others (Figure 2B,D,F; Supplemental Tables S9 and S10).

**FIGURE 2:**
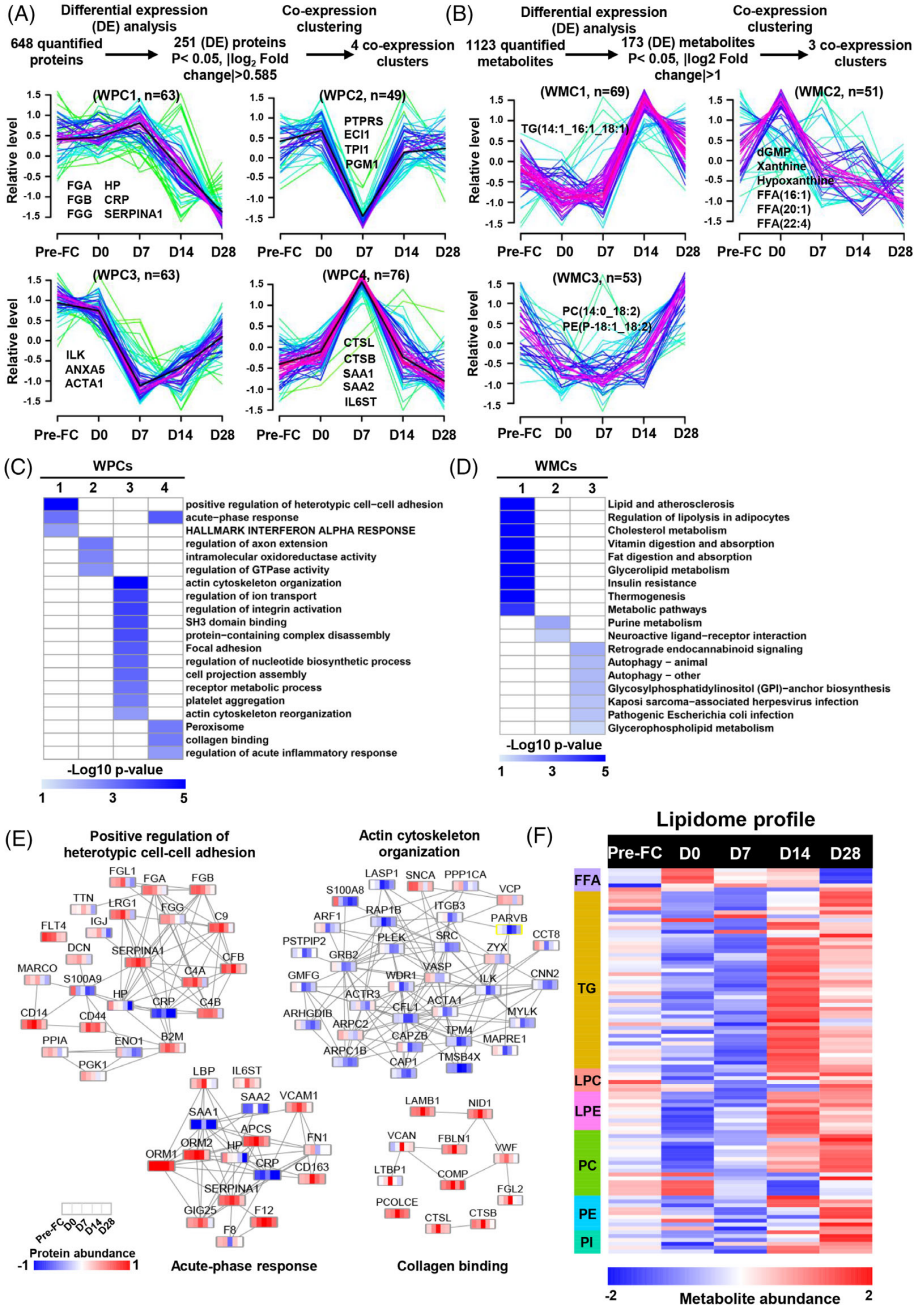
Temporal profiling of proteome and metabolome during CAR‐T cell therapy. (A) Overview of analysis for plasma proteome in B‐ALL patients. The DEPs were assigned to four whole proteome clusters (WPCs) on the basis of Mfuzz clustering analysis. Each line indicates the relative abundance of each protein and is colour‐coded by the cluster membership. Selective proteins in each WPC are shown. (B) Overview of analysis for plasma metabolome in B‐ALL patients. The DEMs were assigned to three whole metabolome clusters (WMCs) according to Mfuzz clustering analysis. Each line indicates the relative abundance of each metabolite and is colour‐coded by the cluster membership. Selective metabolites in each WMC are shown. (C) The heatmap shows the functional annotations of WPCs by GO, KEGG, and Hallmark databases. (D) The heatmap displays the functional annotations of WMCs by KEGG. (E) Proteins involved in the positive regulation of heterotypic cell–cell adhesion, actin cytoskeleton organization, acute‐phase response and collagen binding are indicated with their corresponding expression levels at different time points. (F) Lipid profiles of B‐ALL patients are indicated in the heatmap with their corresponding expression levels at the different time points. FFA, free fatty acids; TG, triglyceride; LPC, lysophosphatidylcholine; LPE, lysophosphatidylethanolamine; PC, phosphatidylcholine; PE, phosphatidylethanolamine; PI, phosphatidylinositol.

We next examined the proteomic and metabolomic differences between patients with cytokine storm (CS) and their counterparts without cytokine storm (NCS). Compared with NCS, 10, 9, 70, 62 and 14 DEPs were found in these five groups, respectively (Supplemental Figure S8A). Before fludarabine/cyclophosphamide lymphodepletion, the proteins that were involved in the positive regulation of the apoptotic process were significantly upregulated in CS patients (Supplemental Figure S8B‐C). Acute phase response underwent the most significant change, and most of DEPs were upregulated in CS patients at day 7 post‐infusion (Supplemental Figure S8B‐C). Interestingly, the apolipoproteins (i.e., apolipoprotein C2 (APOC2) and apolipoprotein C3) were upregulated in CS patients at day 14 after CAR‐T cell infusion (Supplemental Figure S8C). In our metabolomic profiling, there were 23, 60, 103, 152 and 24 DEMs in these five groups, respectively (Supplemental Figure S8D). Glycerophospholipid metabolism underwent a significant change at the time point of day 7 (Supplemental Figure S8E), with a lower level of glycerophospholipids in CS patients (Supplemental Figure S8F). DGs and TGs were abundant in CS patients at the time point of day 14 (Supplemental Figure S8F; Supplemental Table S11). Compared with HC, a higher level of APOC2 was observed in CS patients on day 14 (Supplemental Figure S9). The apolipoproteins might regulate TGs accumulation at day 14 post‐infusion.^5^ Numerous metabolites, including glycerophospholipids and amino acids, were lower in CS patients compared with their counterparts at day 28 post‐infusion (Supplemental Figure S8F), suggesting that CS may result in the durable suppression of these metabolites.

To further elucidate the dynamics of plasma proteome and metabolome during CS, we made co‐expression clustering analyses in CS patients. Since IL‐6 is a critical and multifunctional cytokine in CAR‐T cell‐mediated CS, we identified sets of covarying molecules that were implicated in epithelial mesenchymal transition, glutathione metabolism and tryptophan metabolism in concert with IL‐6 (Figure 3A–D), further revealing a potential regulatory crosstalk between tissue remodeling, metabolic reprogramming and IL‐6. The proteins participating in coagulation and anticoagulation were enriched in the three different clusters (Figure 3E). For instance, intercellular adhesion molecule 1 and vascular cell adhesion molecule 1 are secreted by cytokine‐activated endothelia^6^, ^7^ and belong to WPC1 (Figure 3E). IL‐6 regulated tryptophan metabolism^8^ (Figure 3F). Consistently, tryptophan, kynurenine and kynurenic acid levels were significantly increased at the peak of IL‐6 although there was no statistical difference for kynurenic acid (Figure 3G). One patient with CRES had extremely high levels of tryptophan, kynurenic acid and kynurenine. Kynurenic acid modulates cognition and behaviour by antagonizing the α7‐nicotinic receptor,^9^ suggesting a potential mechanism of CRES.

**FIGURE 3:**
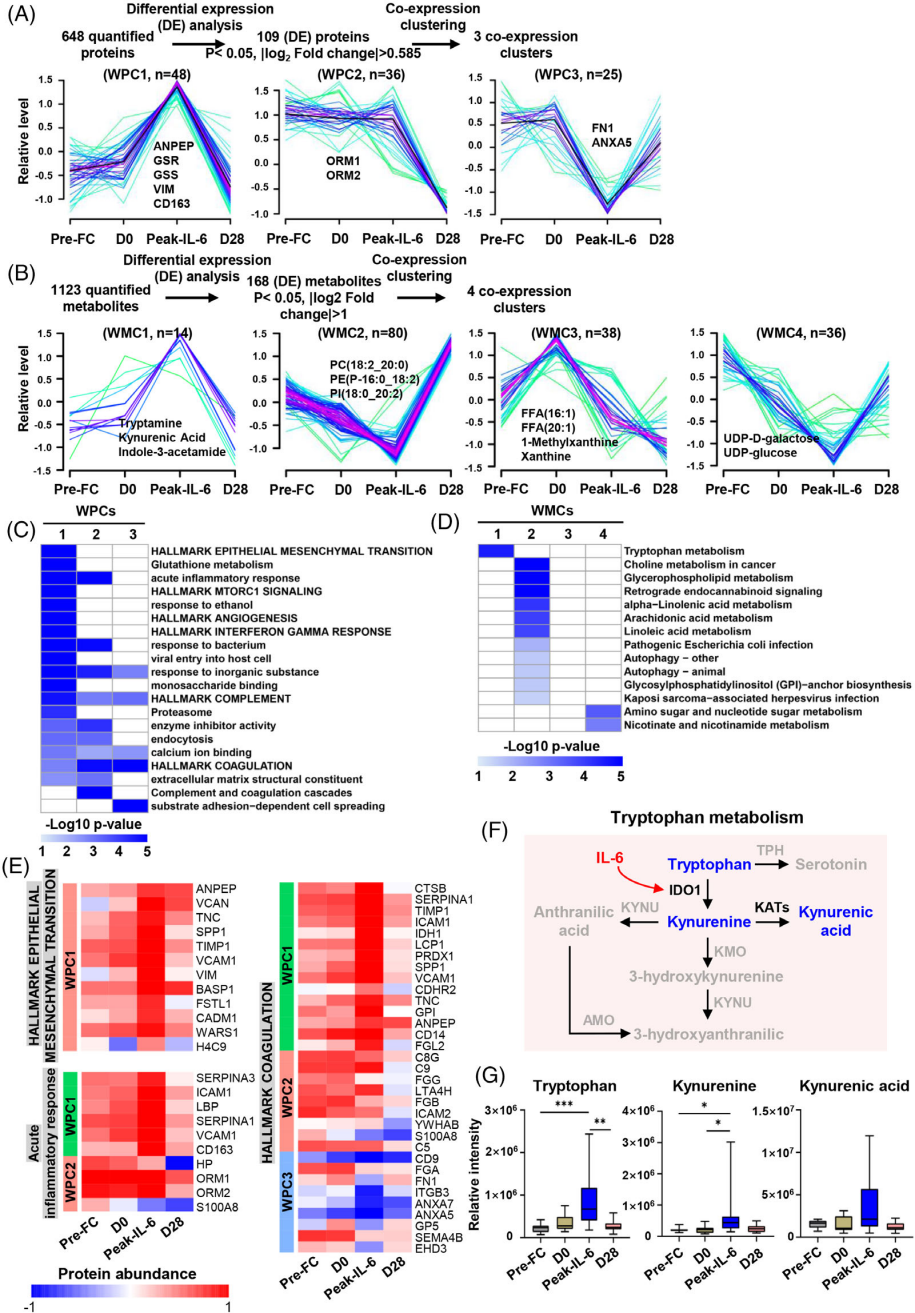
Temporal profiling of proteome and metabolome during cytokines storm. (A) Overview of analysis of plasma proteome in B‐ALL patients with cytokines storm. The DEPs were assigned to three WPCs according to Mfuzz clustering analysis. Each line indicates the relative abundance of each protein and is colour‐coded by the cluster membership. Selective proteins in each WPC are shown. (B) Overview of analysis of plasma metabolome in B‐ALL patients with cytokines storm. The DEMs were assigned to four WMCs according to Mfuzz clustering analysis. Selective metabolites in each WPC are shown. (C) The heatmap exhibits the functional annotations of WPCs by GO, KEGG and Hallmark databases. (D) The heatmap shows the functional annotations of WMCs by KEGG. (E) Proteins involved in the epithelial mesenchymal transition, acute inflammatory response and coagulation are indicated with their corresponding expression levels at the four time points. (F) The schematic illustration depicts the tryptophan metabolism pathway in response to IL‐6. (G) Plasma levels of tryptophan, kynurenine and kynurenic acid were compared at different time points. Peak‐IL‐6 represents the time point when the samples were collected at the peak of IL‐6 level from patients with cytokine storm. The comparisons were made by using a nonparametric test (*p < 0.05, **p < 0.01).

Next, we compared the proteomic and metabolomic differences between sCR patients (achieved stable CR) and non‐CR patients (failed to achieve CR or suffered a relapse). Compared with non‐CR patients, 44, 16, 19, 42 and 68 DEPs were found in these five groups, respectively (Figure 4A). Before lymphodepletion, multiple allograft rejection proteins (i.e. beta‐2‐microglobulin, cathepsin S, inducible T cell co‐stimulator ligand) were downregulated in sCR patients (Figure 4B,C). Meaningfully, DEPs between sCR_D28 and non‐CR_D28 were highly enriched in the pentose phosphate pathway and upregulated in sCR patients (Figure 4B,D). The upregulated pentose phosphate pathway provides the nucleotides and amino acids for cell proliferation,^10^ possibly facilitating immune reconstitution. Compared with non‐CR patients, there were 26, 15, 3, 6 and 4 DEMs in these five groups, respectively (Figure 4E). Before lymphodepletion, DEMs were highly enriched in thermogenesis, many of which were upregulated in non‐CR patients (Figure 4F).

**FIGURE 4:**
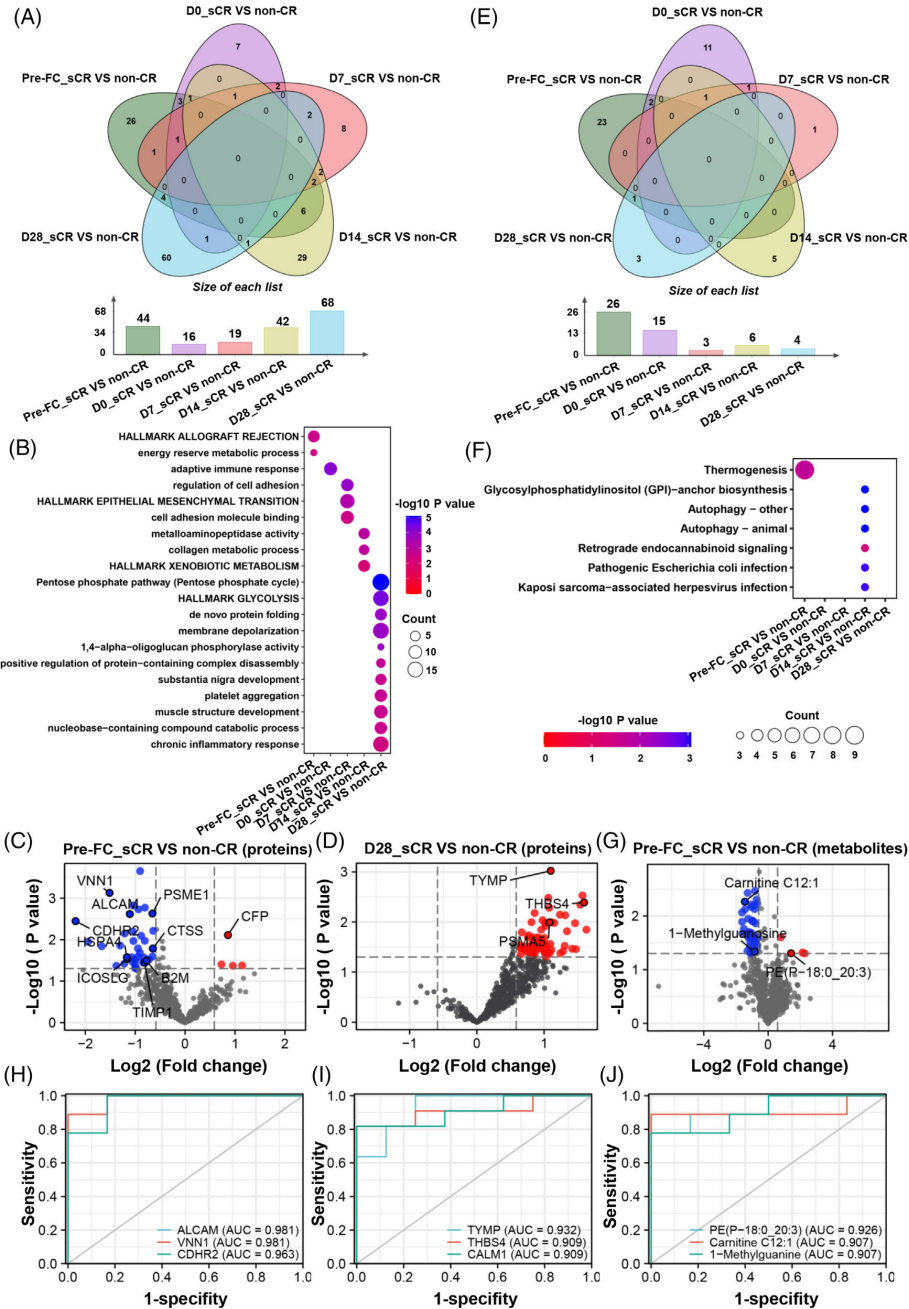
Identification of potential biomarkers for the prognosis of B‐ALL patients receiving humanized anti‐CD19‐CAR‐T cell therapy. (A) The Venn diagrams and the histograms display the numbers of DEPs between sCR patients (achieved stable complete remission (CR) after CAR‐T cell therapy) and non‐CR patients (failed to achieve CR or suffered a relapse after CAR‐T cell therapy or CAR‐T therapy bridging to haematopoietic stem cell transplantation) at different time points. (B) Pathway enrichment analyses of DEPs in term of biological processes are presented in the two bubble charts. (C‐D) The volcano plot shows the protein alterations between sCR patients and non‐CR patients at the time point of pre‐FC and Day 28. (D) The volcano plot shows the protein alterations between the sCR group and non‐CR group at the time point of Day 28. (E) The Venn diagrams and the histograms display the numbers of DEMs between sCR patients and non‐CR patients at different time points. (F) Pathway enrichment analyses of DEMs in term of biological processes are presented in the two bubble charts. (G) The volcano plot shows the protein alterations between sCR patients and non‐CR patients at the time point of Day 28. (H) The receiver operator characteristic curve (ROC) curve analysis displays the predictive power of activated leukocyte cell adhesion molecule (ALCAM), vanin 1 (VNN1) and cadherin‐related family member 2 (CDHR2) for distinguishing sCR patients from non‐CR patients at the time point of pre‐FC. (I) The ROC curve analysis shows the predictive power of thymidine phosphorylase (TYMP), thrombospondin‐4 (THBS4) and calmodulin‐1 (CALM1) for distinguishing sCR patients from non‐CR patients at the time point of Day 28. (J) The ROC curve analysis shows the predictive power of PE (P‐18:0_20:3), carnitine C12:1 and 1‐methylguanine for distinguishing sCR patients from non‐CR patients at the time point of pre‐FC.

We further analyzed longitudinal alterations of plasma proteome and metabolome in patients with different clinical outcomes. The co‐expression clusters were identified in the two groups. As shown in Supplemental Figure S10, the inflammatory response was induced after CAR‐T cell infusion in both groups, while regulation of cytoskeleton organization and actin filament‐based process were downregulated in sCR patients and non‐CR patients, respectively. However, proteins, which were involved in pathways (i.e., spinocerebellar ataxia, pentose phosphate pathway and regulation of the DNA metabolic process), continually dropped in the non‐CR group after CAR‐T cell infusion (Supplemental Figure S10). In our metabolomic profiling, the pathway, including retrograde endocannabinoid signalling, linoleic acid metabolism, choline metabolism in cancer and glycerophospholipid metabolism, were downregulated in both two groups during CAR‐T cell therapy (Supplemental Figure S11). However, the metabolites, which were enriched in multiple pathways (i.e., thermogenesis, sphingolipid metabolism, neurotrophin signalling pathway), continued to decrease in non‐CR patients after CAR‐T cell infusion (Supplemental Figure S11).

Next, we directly used the relative abundance values of proteins and metabolites to identify potential biomarkers for classifying B‐ALL patients with different therapeutic responses (Figure 4H–J). Our proteomic profiling revealed that activated leukocyte cell adhesion molecule (ALCAM), vanin 1 (VNN1) and cadherin‐related family member 2 (CDHR2) were downregulated in sCR patients and showed high accuracy for distinguishing sCR patients from non‐CR patients (Figure 4C,H). Furthermore, thymidine phosphorylase (TYMP), thrombospondin‐4 (THBS4) and calmodulin‐1 (CALM1) were upregulated in sCR patients at day 28 after CAR‐T cell infusion and also exhibited high accuracy in distinguishing patients with different therapeutic responses (Figure 4D,I). Our receiver operator characteristic curve (ROC) analyses found that three plasma metabolites could highly discriminate sCR patients from non‐CR patients, including PE (P‐18:0_20:3), carnitine C12:1 and 1‐methylguanine (Figure 4J). Together, we identified some potential prognostic biomarkers that could distinguish patients with different therapeutic responses.

The limitations should be cautiously considered. First, our study was limited by the small sample size and the specimens were collected at a single center. Second, the roles of altered proteins and metabolites in B‐ALL patients receiving CAR‐T cell therapy need to be elucidated or experimentally validated. For instance, cytoskeleton organization proteins were significantly downregulated in B‐ALL patients. Given that all patients suffered from cytopenia over the course of CAR‐T cell therapy, we infer that dysregulated actin cytoskeleton proteins may correlate with cytopenia. Moreover, various drugs administered to the patients, including prior chemotherapeutic agents, could impact plasma proteome and metabolome. Finally, our results need to be further verified by the studies recruiting more patients with features identical to our cohort of B‐ALL patients.

In summary, this is the first systematic analysis of plasma proteomic and metabolomic alteration of patients with B‐ALL on CAR‐T cell therapy. We revealed how this novel treatment approach works on the human body. Proteomic and metabolomic testing of blood samples would be worthwhile to identify patients at high risk for adverse events and unfavourable prognosis, which facilitates clinical decisions for CAR‐T cell therapy.

## CONFLICT OF INTEREST STATEMENT

Wei Xiong is an employee of Wuhan Sian Medical Technology Co., Ltd. The remaining authors declare that they have no competing interests.

## Supporting information

Supporting Information

Supporting Information

Supporting Information

Supporting Information

Supporting Information

Supporting Information

Supporting Information

Supporting Information

Supporting Information

Supporting Information

Supporting Information

Supporting Information
