# Supplementary material for: Plasma proteomic and metabolomic signatures of B‐ALL patients during CAR‐T cell therapy
Source: Clin Transl Med. 2023 Mar 20;13(3):e1225. doi: 10.1002/ctm2.1225 (PMC10026086; doi:10.1002/ctm2.1225)
Supplement: Supplementary file 1 — Supporting Information [file CTM2-13-e1225-s007.doc]

**Plasma proteomic and metabolomic signatures of B-ALL patients during CAR-T cell therapy**

**Authors:**

Jianghua Wu^1, 2, #^, Lu Tang^1, 2, #^, Mengyi Du^1, 2, #^, Chenggong Li^1, 2^, Haiming Kou^1, 2^, Huiwen Jiang^1, 2^, Wenjing Luo^1, 2^, Yinqiang Zhang^1, 2^, Zhongpei Huang^1, 2^, Danying Liao^1, 2^, Wei Xiong^2^, Heng Mei^1, 2, *^, Yu Hu^1, 2, *^

**Materials and methods**

**Study design and participants**

The study aimed to assess plasma proteomic and metabolomic profiling in patients with B-ALL during humanized anti-CD19-CAR-T cell therapy. In this study, we designed a second-generation humanized CAR, mainly consisting of humanized CD19 single chain variable fragment, CD3ζ transmembrane domain and 4-1BB costimulatory domain. According to clinical trial requirements (NCT04008251), patients were enrolled for humanized anti-CD19-CAR-T cell therapy if they had relapsed or were refractory to their previous treatments, including autologous or allogenic hematopoietic stem cell transplantation (HSCT). Between September 1, 2019, and February 28, 2021, 24 CD19-positive B-ALL patients were recruited into the study at Union Hospital, Tongji Medical College, Huazhong University of Science and Technology. Because specimens from 4 patients were not preserved, we obtained plasma samples from 20 CD19-positive relapsed/refractory B-ALL patients for proteomic and metabolomic profiling. The patients received lymphodepleting regimens of fludarabine 30 mg/m^2^ for three days and cyclophosphamide 750 mg/m^2^ for one day, and then received the infusion of the humanized anti-CD19 CAR-T cells at a median dose of 2.0x10^6^ (range 2.0 x10^6^-4.0 x10^6^) CAR-T cells per kilogram. All patients were followed up until they died, were lost to follow-up, or withdrew consent. Adverse events, including CAR-T-cell-related encephalopathy syndrome (CRES), were evaluated according to the National Cancer Institute Common Terminology Criteria for Adverse Events (CTCAE) version 4.03. CRS was graded according to MSKCC CRS grading system. Clinical information of patients was collected from the hospital electronic history system. 22 age- and gender-matched healthy controls (HC) were recruited from laboratory workers and medical students at Union Hospital. The study was approved by Ethics Committee of Union Hospital, Tongji Medical College, Huazhong University of Science and Technology (REC ref no. [2019]008). Informed consent was obtained from each subject in accordance with the Declaration of Helsinki.

**Sample collection and extraction**

Blood samples were collected in EDTA-treated tubes from each subject. The blood sample was obtained and immediately centrifuged at 600 × g for 10 min for plasma separation. The plasma was stored at −80°C until further analysis.

**Sample preparation and TMT-labeling**

10 μl of plasma were mixed with 190 μl reaction solution (1% SDC, 10 mM TCEP, and 40 mM CAA). The reaction was set at 60 °C for 30 min for protein denaturation, disulfide bond reduction, and cysteine -SH alkylation. The concentration of protein was measured by Bradford method. The samples were diluted with equal volume of H2O. Trypsin was added into the diluted samples at a ratio of 1:50 (enzyme: protein, w/w) for overnight digestion at 37 °C. After centrifugation (12,000 g, 15 min), peptide purification was performed by self-made desalting columns filled with Poly (styrene-divinylbenzene) copolymer materials as described^1^. The purified peptide was stored at -20°C for later use.

Tandem mass tag (TMT) labeling was performed according to manufacturer’s instructions. Briefly, peptides were reconstituted in TMT reagent buffer, and were separately labeled with different TMT labeling reagents. The internal reference sample pooled from all the 123 samples was labeled using channel 126 for each batch of TMT labeling experiment, and was used as the quality control. The labeled samples were then mixed and subjected to Sep-Pak C18 desalting. The labeling efficiency of each labeled mixture was examined by mass spec identification of 2 mg of the mixture with TMT (N-terminal/K) as variable modifications. The labeling efficiency must pass the threshold of 95% before proceeding to the fractionation step. The remaining mixture for each group of TMT experiment was fractionated by using high pH reverse phase chromatography into 60 fractions and further concatenated into 20 fractions (by combining of fractions 1, 21 and 41; fractions 2, 22 and 42; and so on). Each fraction was vacuum-dried and stored at -80°C until MS analysis^2^.

**Proteome LC-MS/MS analysis**

LC-MS/MS data acquisition was performed on a Orbitrap Exploris 480 mass spectrometer coupled with an Easy-nLC 1200 system^3^. For each fraction, peptides were loaded onto auto-sampler and separated in a C18 analytical column (75μm × 25cm, C18, 1.9 μm, 100 Å). Mobile phase A (0.1% formic acid) and mobile phase B (80% ACN, 0.1% formic acid) were used to establish the separation gradient. A constant flow rate was set at a flow of 300 nL/min. For the analysis in data-dependent acquisition mode, each scan cycle is comprised of one full-scan mass spectrum (R = 60 K, AGC = 300%, max IT = 20 ms, scan range = 350-1500 m/z) followed by 20 MS/MS events (R = 15 K, AGC = 100%, max IT = auto, cycle time = 2 s, TurboTMT enabled). HCD collision energy was set to 35. The isolation window for precursor selection was 1.2 Da. Former target ion exclusion was set for 35 s.

**Methods for the extraction of hydrophilic and** **hydrophobic compounds**

For the extraction of hydrophilic compounds, sample was thawed on ice, vortex for 10 s and mix well. 300 μl of 20 % acetonitrile methanol internal standard extractant was added to 50 μl of plasma. Whirl the mixture for 3 min and centrifuge it with 12000 rpm at 4 °C for 10 min. Transfer 200 μL of the supernatant and leave in a refrigerator at -20 °C for 30 min. Then, centrifuge it with 12000 rpm for 3 min. Take 180 μl of supernatant in the liner of the corresponding injection bottle for LC-MS/MS analysis.

For the extraction of hydrophobic compounds, sample was thawed on ice, whirl for 10 s. Take 50 μl of one sample and homogenize it with 1mL of the extraction solvent (MTBE: MeOH = 3:1, v/v) containing internal standard mixture. Whirl the mixture for 15 min. Then add 200 μl of water and whirl the mixture for 1 min, and centrifuge it with 12,000 rpm at 4 °C for 10 min. Extract 200 uL supernatant and concentrate it. Dissolve powder with 200 μl mobile phase B. Finally, take the dissolving solution into the sample bottle for LC-MS/MS analysis.

**ESI-Q TRAP-MS/MS of hydrophilic compounds**

LIT and triple quadrupole (QQQ) scans were acquired on a triple quadrupole-linear ion trap mass spectrometer (QTRAP), QTRAP® LC-MS/MS System, equipped with an ESI Turbo Ion-Spray interface, operating in positive and negative ion mode and controlled by Analyst 1.6.3 software (Sciex). The ESI source operation parameters were as follows: source temperature 500 °C; ion spray voltage 5500 V (positive), -4500 V (negative); ion source gas I, gas II and curtain gas were set at 55, 60, and 25.0 psi, respectively; the collision gas was high. Instrument tuning and mass calibration were performed with 10 and 100 μmol/L polypropylene glycol solutions in QQQ and LIT modes, respectively. A specific set of MRM transitions were monitored for each period according to the metabolites eluted within this period.

**ESI-Q TRAP-MS/MS of hydrophobic compounds**

LIT and triple quadrupole (QQQ) scans were acquired on a triple quadrupole-linear ion trap mass spectrometer (QTRAP), QTRAP® LC-MS/MS System, equipped with an ESI Turbo Ion-Spray interface, operating in positive and negative ion mode and controlled by Analyst 1.6.3 software (Sciex). The ESI source operation parameters were as follows: ion source, turbo spray; source temperature 500 °C; ion spray voltage 5500 V (Positive), -4500 V (Negative); ion source gas 1, gas 2 and curtain gas were set at 45, 55, and 35 psi, respectively; the collision gas was medium. Instrument tuning and mass calibration were performed with 10 and 100 μmol/L polypropylene glycol solutions in QQQ and LIT modes, respectively. QQQ scans were acquired as MRM experiments with collision gas (nitrogen) set to 5 psi. DP and CE for individual MRM transitions was done with further DP and CE optimization. A specific set of MRM transitions were monitored for each period according to the metabolites eluted within this period.

**Database search**

MS raw data were analyzed with MaxQuant (V2.0.1.0) using the Andromeda database search algorithm^4^. The human proteome database contained 20,381 Swiss-Prot/reviewed human protein sequences downloaded from the UniProt database (https://www.uniprot.org/proteomes/UP000005640, on March 12, 2021). The spectra files were searched against the database using the following parameters: Type, TMT; Variable modifications, Oxidation (M), Deamidation (NQ), Acetyl (Protein N-term); Fixed modifications, Carbamidomethyl (C); Digestion, Trypsin/P. The MS1 match tolerance was set as 20 parts per million (ppm) for the first search and 4.5 ppm for the main search; the MS2 tolerance was set as 20 ppm; match between runs was used for identification transfer. Search results were filtered with 1% false discovery rate at both protein and peptide levels. Proteins denoted as decoy hits, contaminants, or only identified by sites were discarded, and the remaining proteins were used for further analysis.

According to the retention time and mass-to-charge ratio, metabolites were identified by using a home-made metadata database of more than 3000 standards. Quantification of plasma metabolites was performed using MultiQuant software package 3.0.2 (Sciex), which integrated and calibrated the chromatographic peaks. To ensure the data quality, we calculated coefficient of variation (CV) values for all metabolites, and removed low-quality hits whose CV values were larger than 0.5.

**Data processing of proteome and metabolome**

For each batch of the plasma proteomic data, the protein abundance in one patient sample was normalized against its corresponding abundance in the pooled sample. The relative protein abundance was used for further analyses across different batches. To guarantee the data quality and effectively use the proteomic data, proteins quantified in < 70% samples were removed. To impute missing values of remaining proteins, the mean and standard deviation (SD) of the distribution of the real intensities were determined, and a new distribution with a downshift of 1.8 SD and width of 0.3 SD was created. The total matrix was imputed using these values, and statistical analyses were then performed. Metabolites with over 50% missing ratios in each group were removed for the metabolomics dataset. Missing values were imputed with the minimal value.

**Functional enrichment analysis**

The pathway enrichment of identified proteins was analyzed by Metascape web-based platform integrating Gene Ontology (GO) biological processes, Kyoto Encyclopedia of Genes and Genomes (KEGG) pathway and Hallmark gene set^5^. All the quantified proteins were set as background and pathways with a count ≥ 3, p-value < 0.01 and enrichment ratio > 1.5 were screened out as enriched pathways. Identified metabolites were annotated using KEGG Compound database (http://www.kegg.jp/kegg/compound/); annotated metabolites were then mapped to KEGG Pathway database (http://www.kegg.jp/kegg/pathway.html). All the quantified metabolites were set as background and pathways with a count ≥ 3 and p-value < 0.05 were screened out as enriched pathways.

**Statistics**

Categorical variables are shown as frequencies or percentages. Comparisons of the data between different groups was evaluated by either two-tailed t-test or a nonparametric test. The criteria for differentially expressed proteins (DEPs) selection was that p value should be less than 0.05 and |log2 fold change| should be larger than 0.585. The differentially expressed metabolites (DEMs) were determined by VIP ≥ 1, p value < 0.05 and |log2 fold change| ≥ 1. The clustering analysis was performed by R package Mfuzz, according to fuzzy c-means clustering algorithm (CM). The statistical analyses were conducted by GraphPad Prism 8 software (GraphPad Software, La Jolla, CA) and R software version 4.0.2 (Institute for Statistics and Mathematics, Vienna, Austria).

**Reference**

1. Rappsilber J, Mann M, Ishihama Y: Protocol for micro-purification, enrichment, pre-fractionation and storage of peptides for proteomics using StageTips. *Nat Protoc.* 2007; 2(8):1896-1906.

2. Batth TS, Francavilla C, Olsen JV: Off-line high-pH reversed-phase fractionation for in-depth phosphoproteomics. *J Proteome Res.* 2014; 13(12):6176-6186.

3. Bekker-Jensen DB, Martinez-Val A, Steigerwald S *et al*: A Compact Quadrupole-Orbitrap Mass Spectrometer with FAIMS Interface Improves Proteome Coverage in Short LC Gradients. *Mol Cell Proteomics.* 2020; 19(4):716-729.

4. Tyanova S, Temu T, Cox J: The MaxQuant computational platform for mass spectrometry-based shotgun proteomics. *Nat Protoc.* 2016; 11(12):2301-2319.

5. Zhou Y, Zhou B, Pache L *et al*: Metascape provides a biologist-oriented resource for the analysis of systems-level datasets. *Nat Commun.* 2019; 10(1):1523.

**Supplementary Figures and Figure legends**


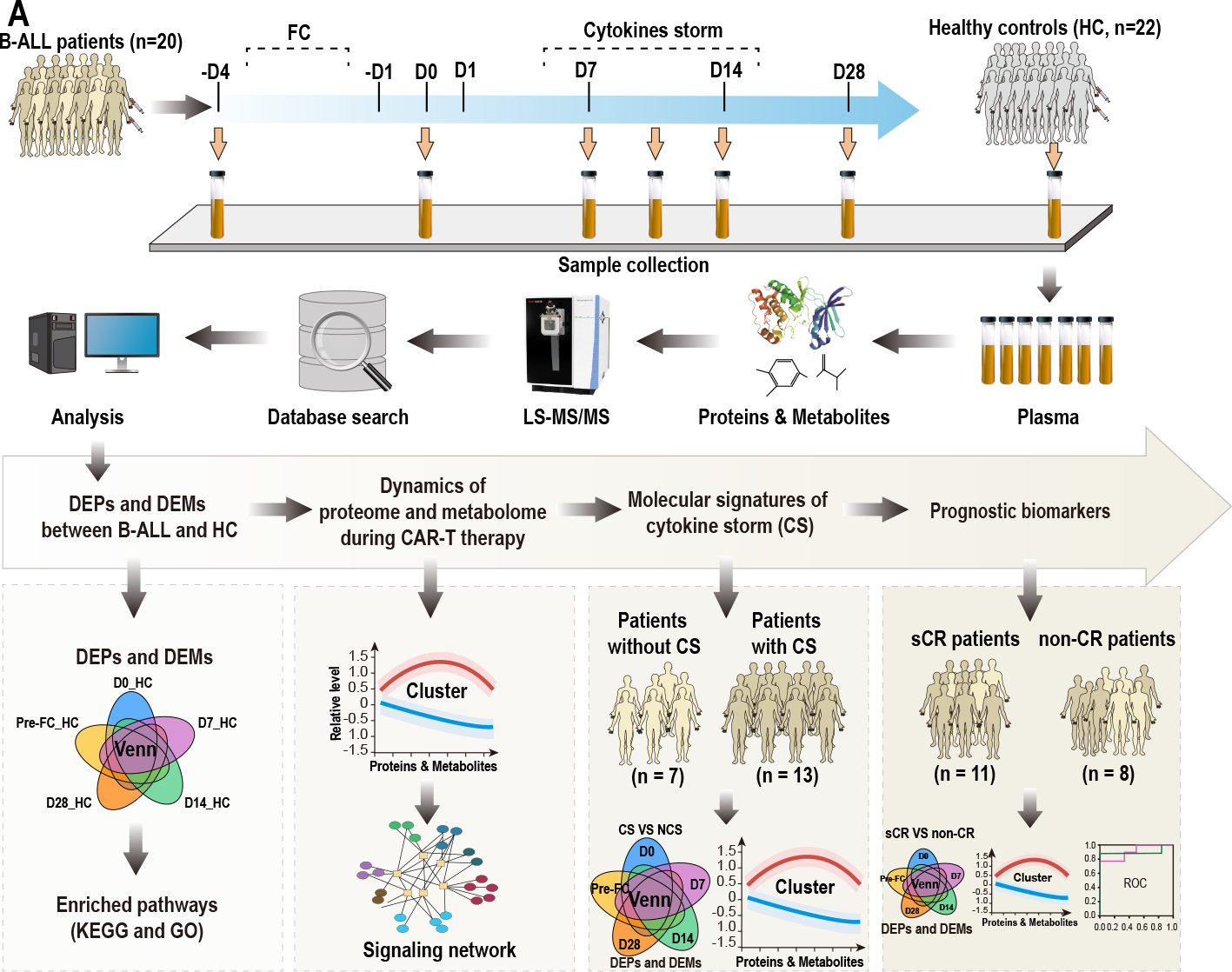


**Figure S1. Workflow of the study.** **(A)** The workflow for processing the proteomic and metabolomic data was shown, including plasma separation, LCMS/MS analysis, database search, and further computational analyses. A four-step analysis process was performed for our data. Blood samples were collected from B-ALL patients at different time points, including before fludarabine/cyclophosphamide lymphodepletion (Pre-FC, n =16), Day 0 (D0, n = 20), Day 7 (D7, n = 20), Day 14 (D14, n = 20), Day 28 (D28, n = 20), and Peak-IL-6 (n = 13). Pre-FC represents the time point when the samples were collected before the treatment of fludarabine/cyclophosphamide (FC) lymphodepletion. Due to incomplete preservation, we obtained 16 plasma samples at the time point of Pre-FC. D0, D7, D14 and D28 represent the time points when the samples were collected on the 0, 7^th^, 14^th^ and 28^th^ day after CAR-T cell infusion. Peak-IL-6 represents the time point when the samples were collected from patients with cytokine storm at the peak of IL-6 level (n = 13), in which 8 samples were obtained on the 7^th^ and 14^th^ day after CAR-T cell infusion. Thus, we in total collected 101 plasma samples from B-ALL patients. 22 healthy controls (HC) were enrolled for comparison. Because 1 patient was lost to follow-up after discharge, we used the data from the remaining 19 patients for prognostic analysis. DEPs, differentially expressed proteins; DEMs, differentially expressed metabolites; sCR represents patients who achieved stable complete remission (CR) after CAR-T cell therapy; non-CR represents patients who failed to achieve CR or suffered a relapse after CAR-T cell therapy.


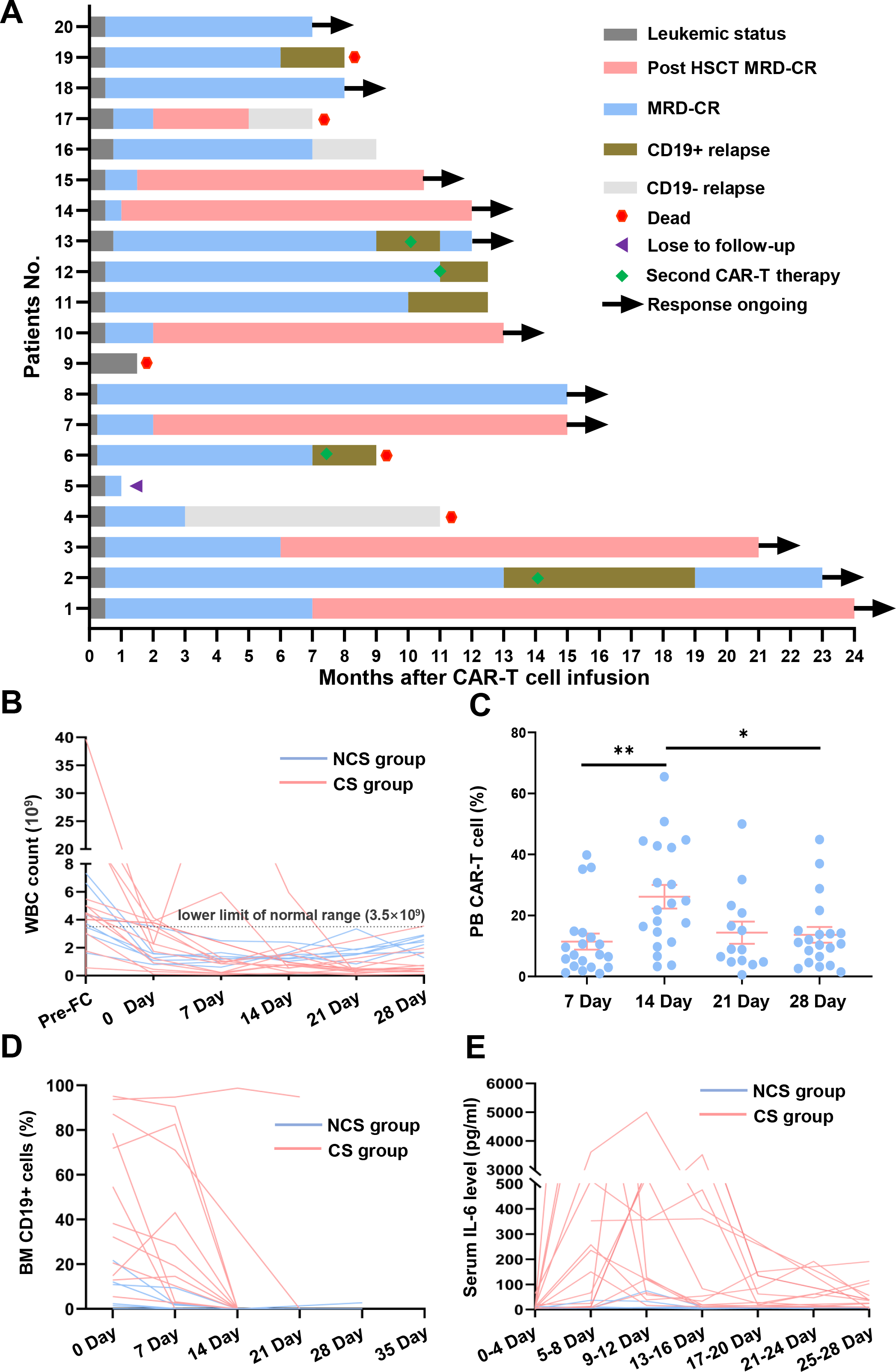


**Figure S2. Responses of patients with B-ALL to humanized anti-CD19-CAR-T cell therapy. (A)** A total of 20 patients diagnosed with resistant or refractory CD19+ B-ALL received an infusion of humanized anti-CD19-CAR-T cells. Responses over time of each patient are presented as a swimmer plot. **(B)** The count of white blood cell (WBC) over the course of CAR-T cell therapy. Pre-FC represents the time point when the data was collected before the fludarabine/cyclophosphamide lymphodepletion. **(C)** The percentages of peripheral blood (PB) CAR-T cells in lymphocytes were flow cytometrically determined and presented in a scatter plot. The comparisons were determined by one-way ANOVA, followed by Bonferroni`s multiple comparison test (*p < 0.05, **p < 0.01). **(D)** The dynamic changes of the percentages of bone marrow (BM) CD19+ cells in lymphocytes are determined by flow cytometry and presented in a line chart. **(E)** The dynamic changes of the level of serum IL-6 after CAR-T cell infusion in each patient are presented in a line chart. CS group represents patients with cytokine storm (n =13), whereas NCS group denotes patients without cytokine storm (n = 7). MRD-CR represents patients who achieved complete remission (CR) and their results of minimal residual disease (MRD) were negative after CAR-T cell therapy; Post HSCT MRD-CR represents patients who achieved CR and their results of minimal residual disease (MRD) were negative after CAR-T therapy bridging to hematopoietic stem cell transplantation (HSCT); Cytokine release syndrome was graded according to the American Society for Transplantation and Cellular Therapy grading criteria.


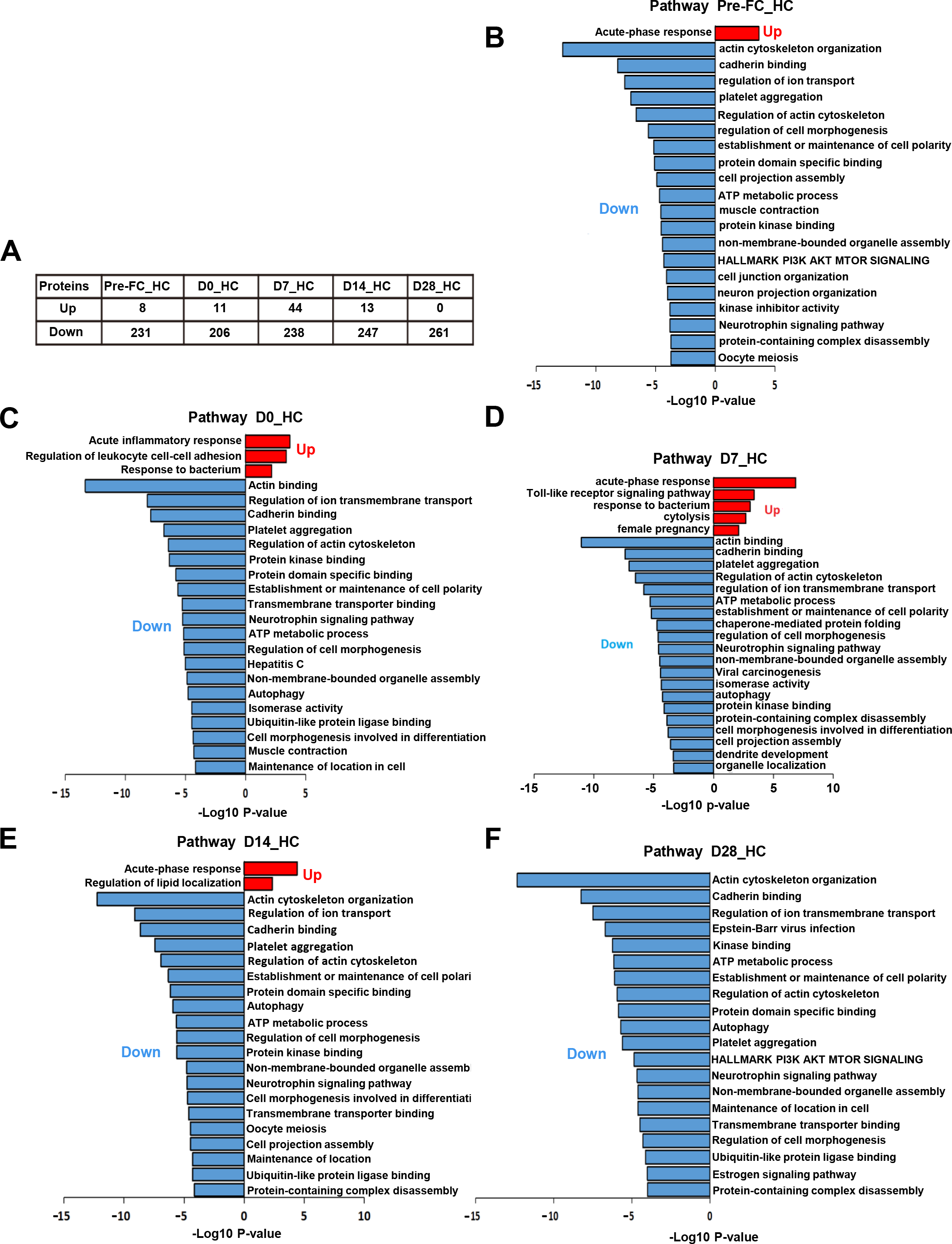


**Figure S3. Alteration of plasma proteome in patients with B-ALL.** **(A)** The table displays the numbers of upregulated and downregulated differentially expressed proteins (DEPs) in the comparisons of Pre-FC versus HC, D0 versus HC, D7 versus HC, D14 versus HC, and D28 versus HC. **(B-F)** Upregulated (red) and downregulated (blue) pathways based on enrichment analyses of DEPs in term of biological processes are presented in the comparisons of Pre-FC versus HC, D0 versus HC, D7 versus HC, D14 versus HC and D28 versus HC, respectively.


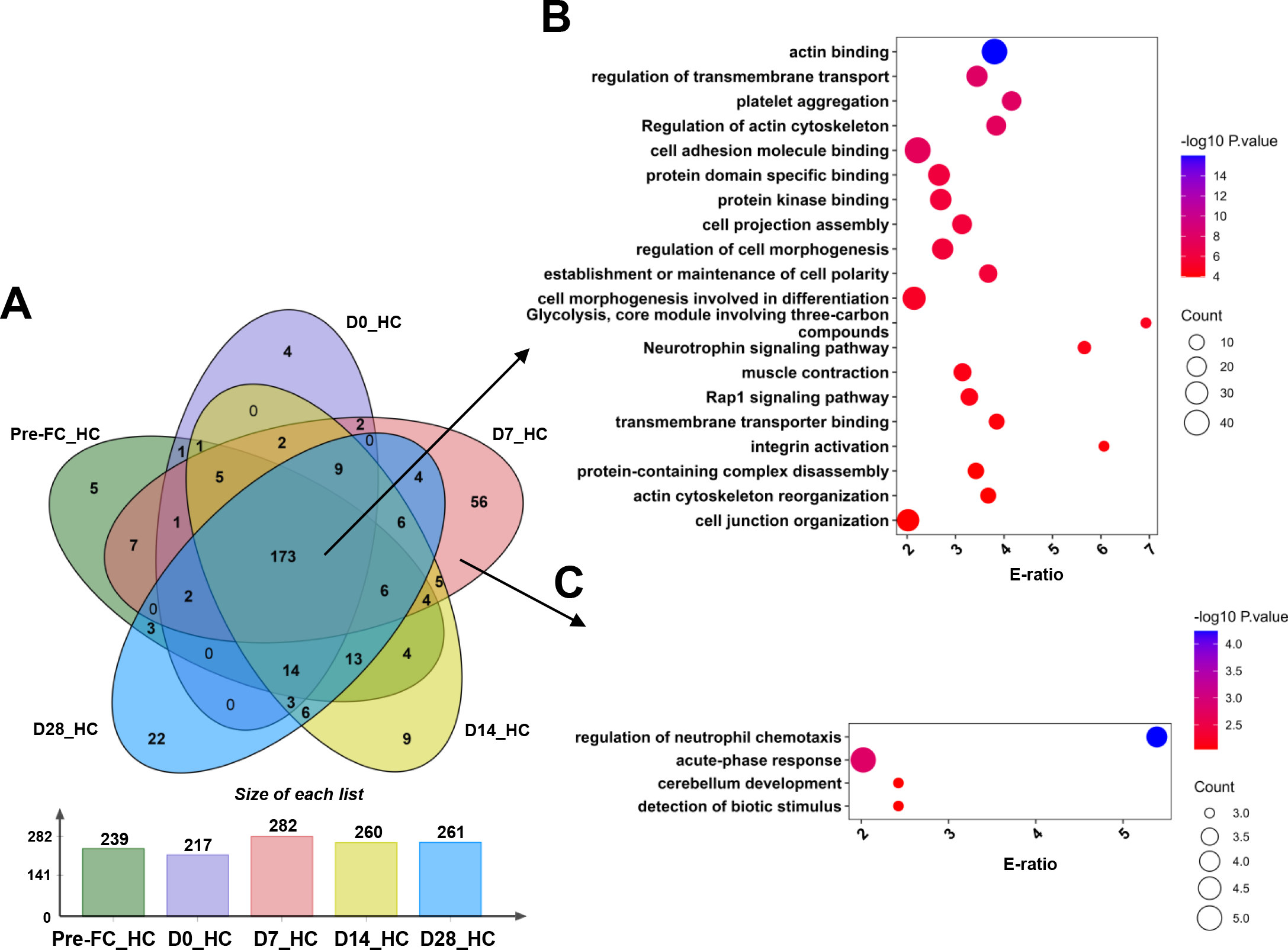


**Figure S4. Plasma proteomic characteristic in patients with B-ALL during CAR-T therapy. (A)** The Venn diagram and the histogram display the numbers of differentially expressed proteins (DEPs) in the comparisons of Pre-FC versus HC, D0 versus HC, D7 versus HC, D14 versus HC and D28 versus HC. **(B)** Pathway enrichment analyses of 173 DEPs shared by five groups in term of biological processes are presented in the bubble chart. **(C)** Pathway enrichment analyses of 56 DEPs specifically expressed on the 7^th^ day after CAR-T cell infusion are presented in the bubble chart.


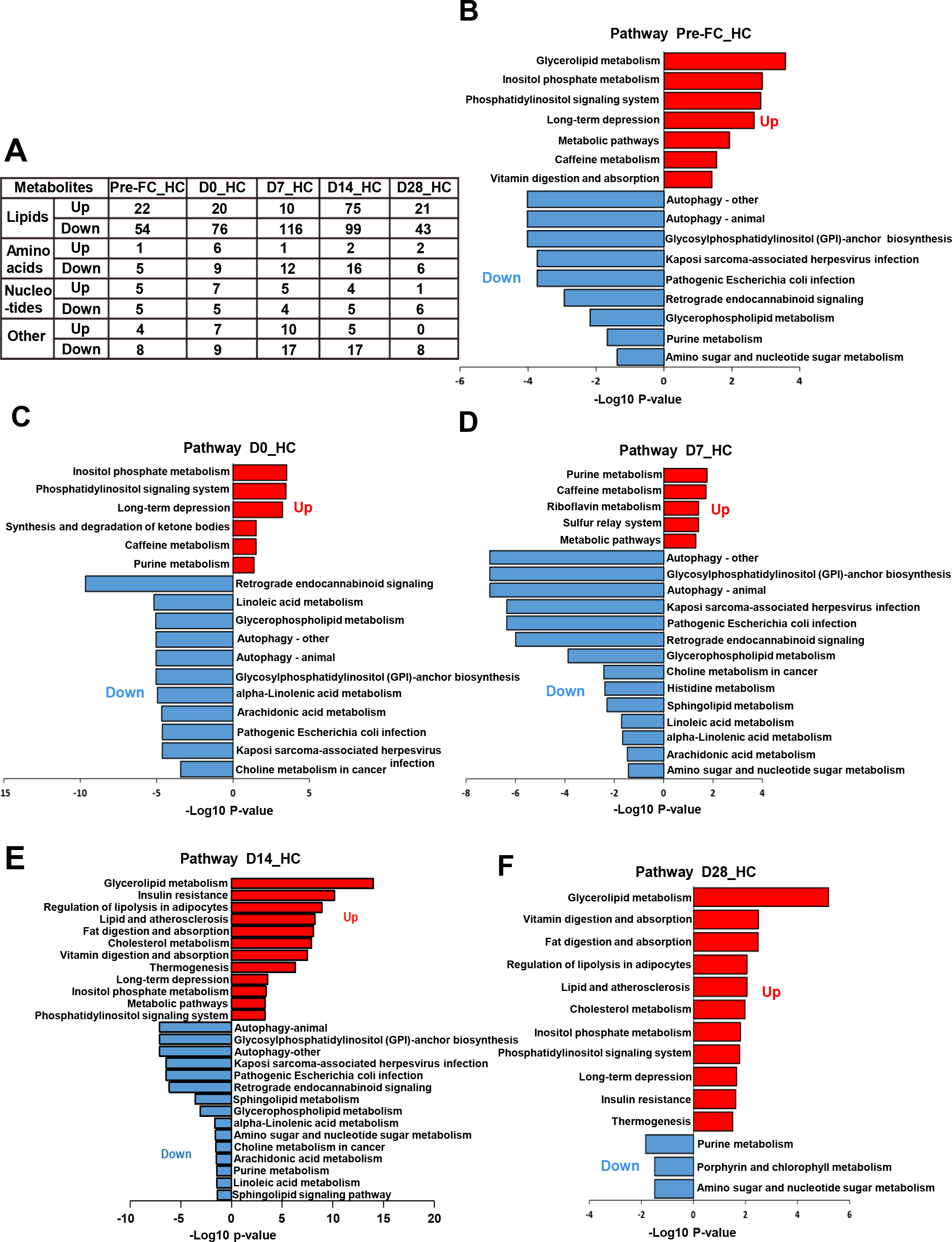


**Figure S5. Alteration of plasma metabolome in patients with B-ALL.** **(A)** The table displays the numbers of upregulated and downregulated differentially expressed metabolites (DEMs) in the comparisons of Pre-FC versus HC, D0 versus HC, D7 versus HC, D14 versus HC, and D28 versus HC. **(B-F)** Upregulated (red) and downregulated (blue) pathways based on enrichment analyses of DEMs in term of biological processes are presented in the comparisons of Pre-FC versus HC, D0 versus HC, D7 versus HC, D14 versus HC and D28 versus HC, respectively.


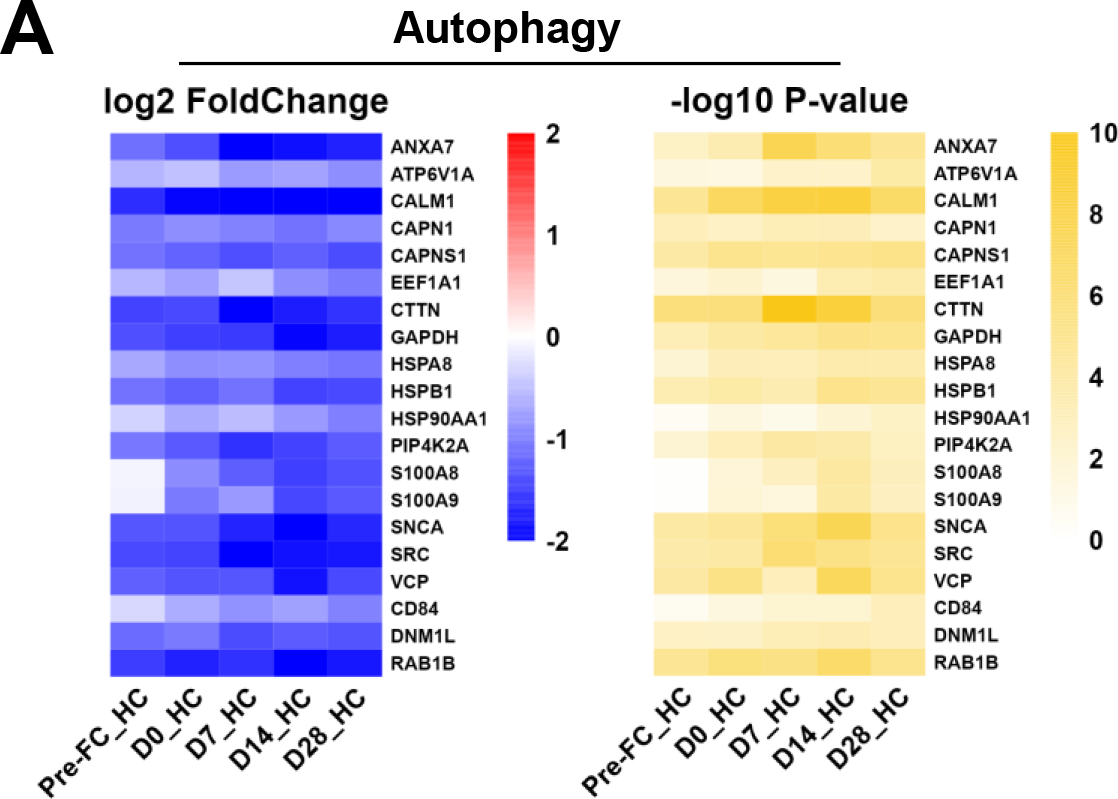


**Figure S6. Dysregulated autophagy in patients with B-ALL. (A)** Plasma levels of proteins in each group in relation to healthy control and the associated p values in the terms of autophagy.


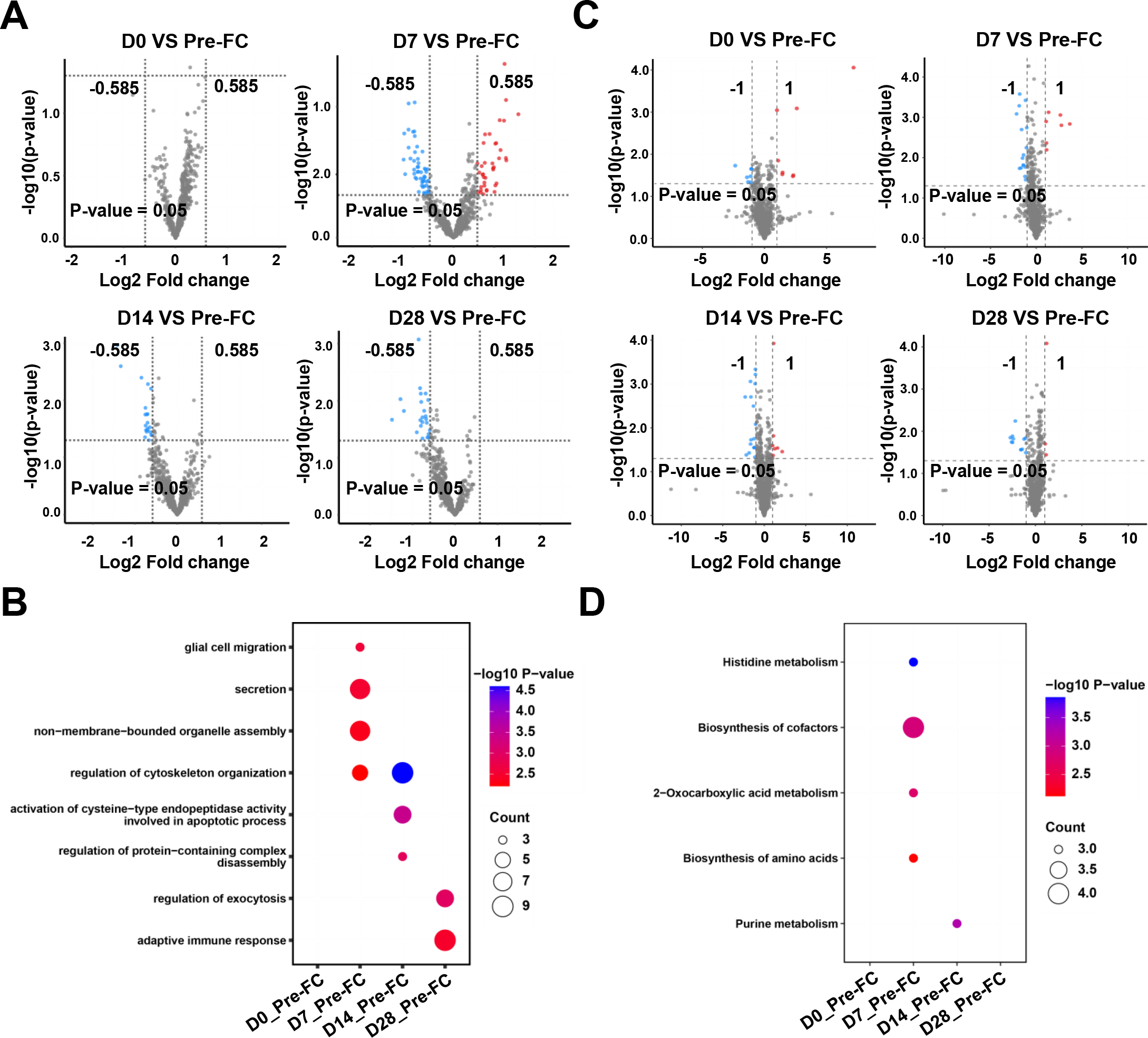


**Figure S7. The differences in plasma molecular signatures in B-ALL patients compared with pretreatment samples. (A)** The Volcano plot shows the proteomic alterations between B-ALL patients (Day-0, Day-7, Day-14, and Day-28) compared with pretreatment samples. **(B)** Pathway enrichment analyses of DEPs in term of biological processes are presented in the bubble chart. **(C)** The Volcano plot shows the metabolomic alterations between B-ALL patients (Day-0, Day-7, Day-14, and Day-28) compared with pretreatment samples. **(D)** Pathway enrichment analyses of DEMs in term of biological processes are presented in the bubble chart.


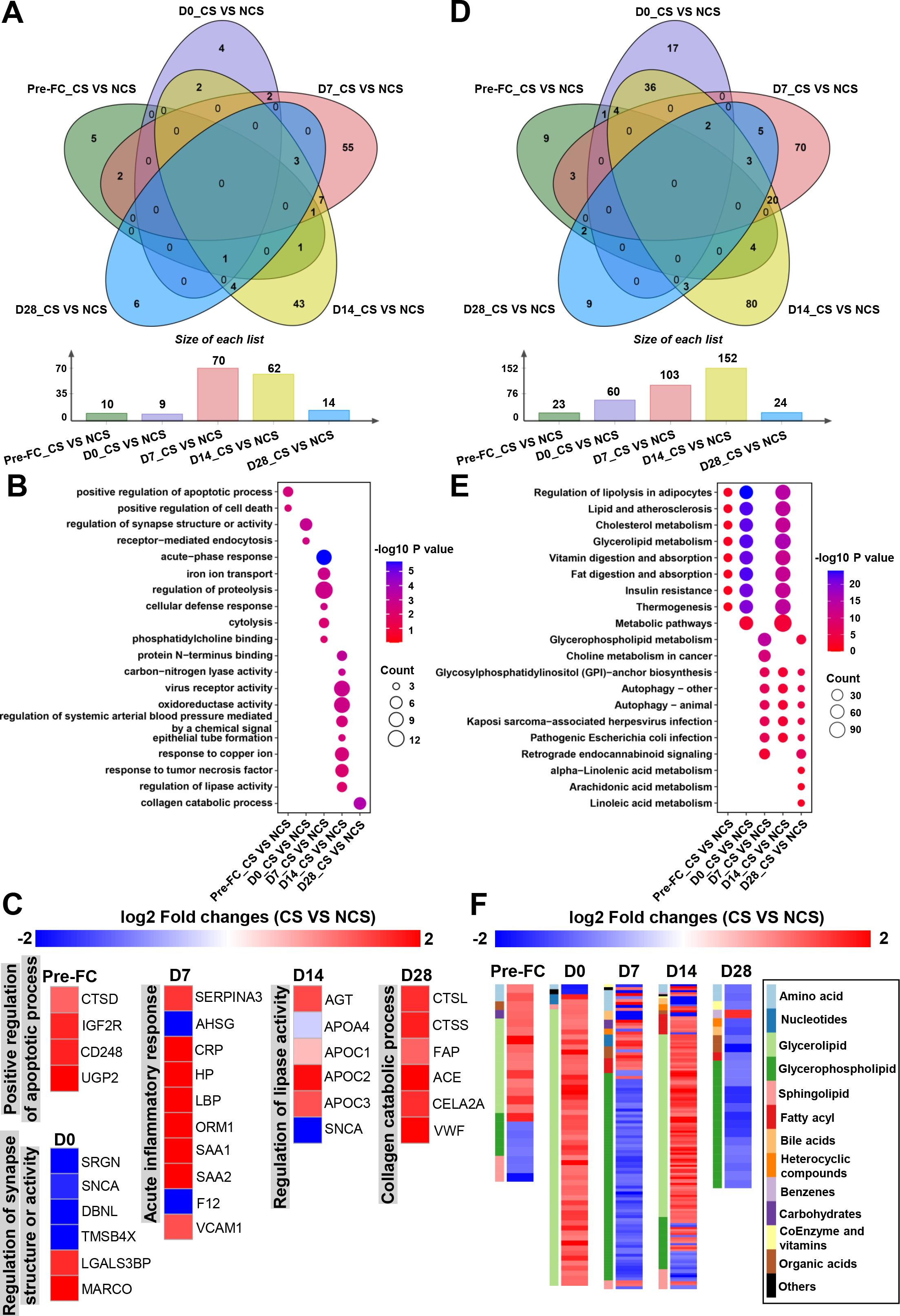


**Figure S8. Plasma proteomic and metabolomic profiles differ between** **patients with cytokines storm (CS) and their counterparts without cytokines storm (NCS). (A)** The Venn diagram and the histogram display the number of differentially expressed proteins (DEPs) between patients with cytokines storm (CS) and patients without cytokines storm (NCS) at different time points. (**B**) Pathway enrichment analyses of DEPs in term of biological processes are presented in the bubble chart. (**C**) The values of the log of fold changes (CS versus NCS) of proteins in the selected pathways are shown at the different time points. **(D)** The Venn diagram and the histogram display the number of differentially expressed metabolites (DEMs) between CS group and NCS group at different time points. (**E**) KEGG-based enrichment analysis of DEMs in term of biological processes is presented in the bubble chart. (**F**) The values of the log of fold changes (CS versus NCS) of metabolites are shown at the different time points.


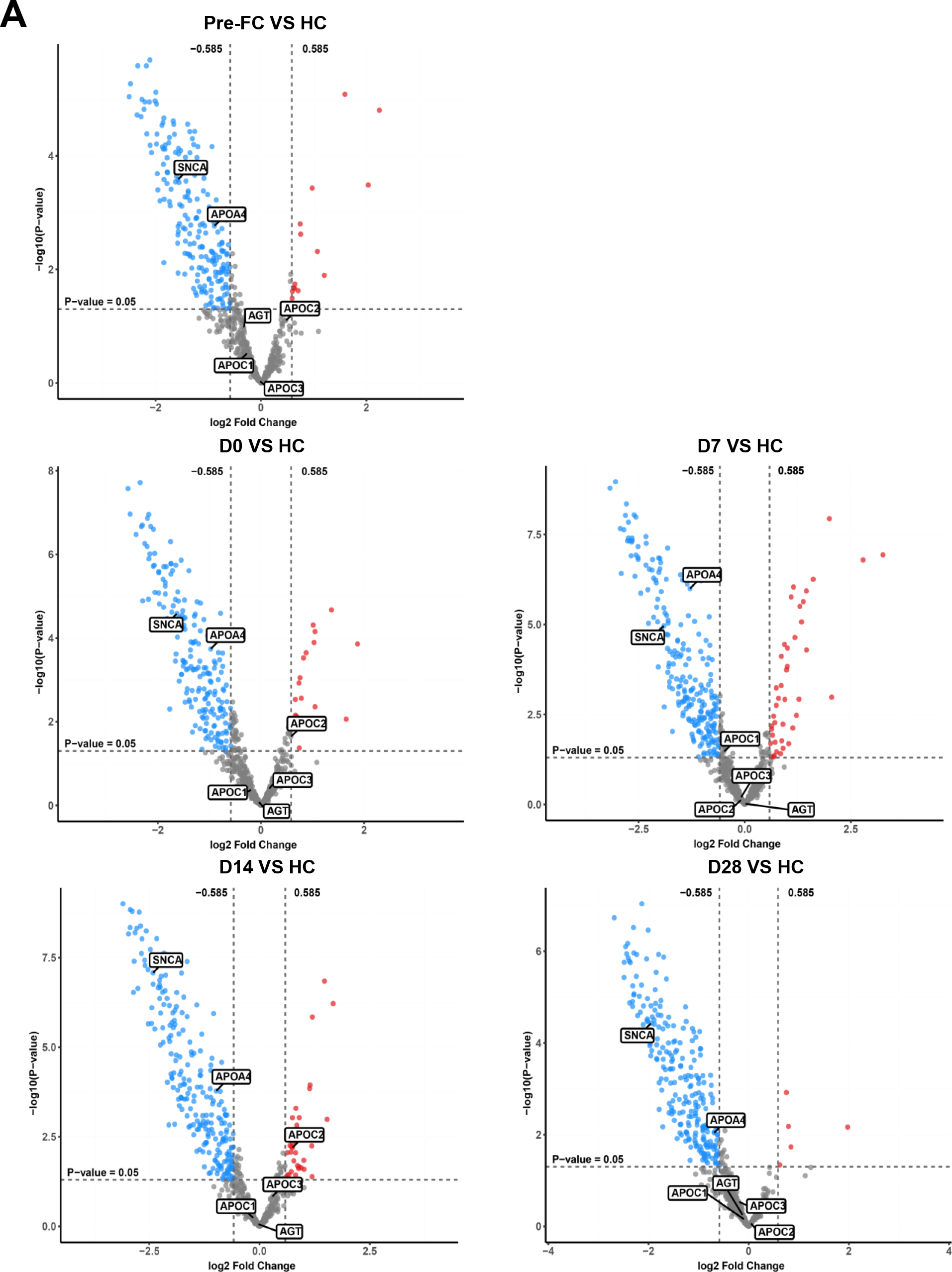


**Figure S9. The expression differences of proteins that were involved in the regulation of lipid localization** **between B-ALL patients with cytokines storm and HC. (A)** The Volcano plot shows the expression differences of proteins that were involved in the regulation of lipid localization between B-ALL patients with cytokines storm at the different timepoints and healthy controls (HC).


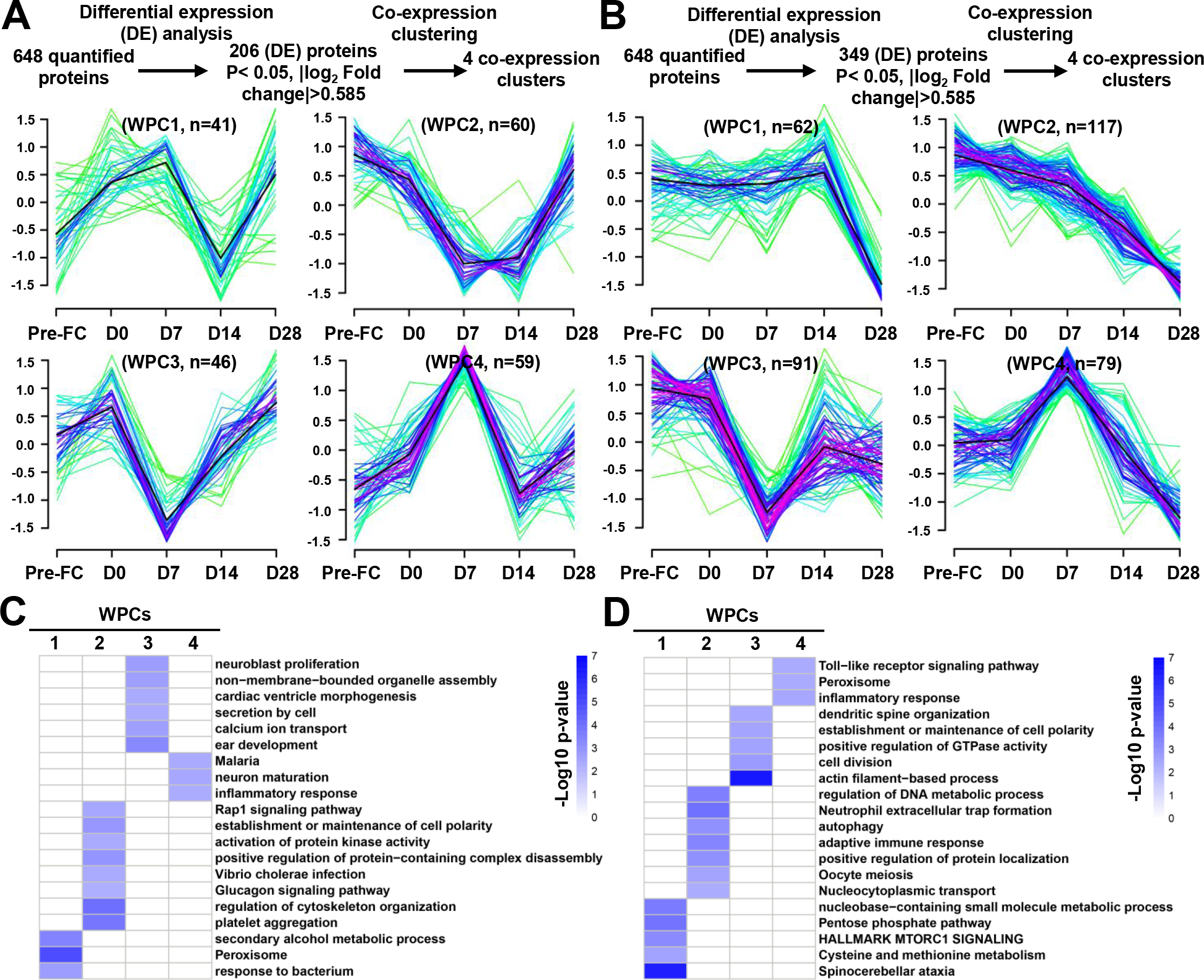


**Figure S10.** **Temporal profiling of proteome during CAR-T cell therapy.** (**A)** Overview of analysis for plasma proteome in sCR patients. The differentially expressed proteins (DEPs) were assigned to four whole proteome clusters (WPCs) on the basis of Mfuzz clustering analysis. Each line indicates the relative abundance of each protein and is color-coded by the cluster membership. **(B)** Overview of analysis for plasma proteome in non-CR patients. The DEPs were assigned to four WPCs according to Mfuzz clustering analysis. Each line indicates the relative abundance of each proteins and is color-coded by the cluster membership. **(C)** The heatmap shows the functional annotations of WPCs by GO, KEGG, and Hallmark databases in sCR patients. **(D)** The heatmap shows the functional annotations of WPCs by GO, KEGG, and Hallmark databases in non-CR patients.


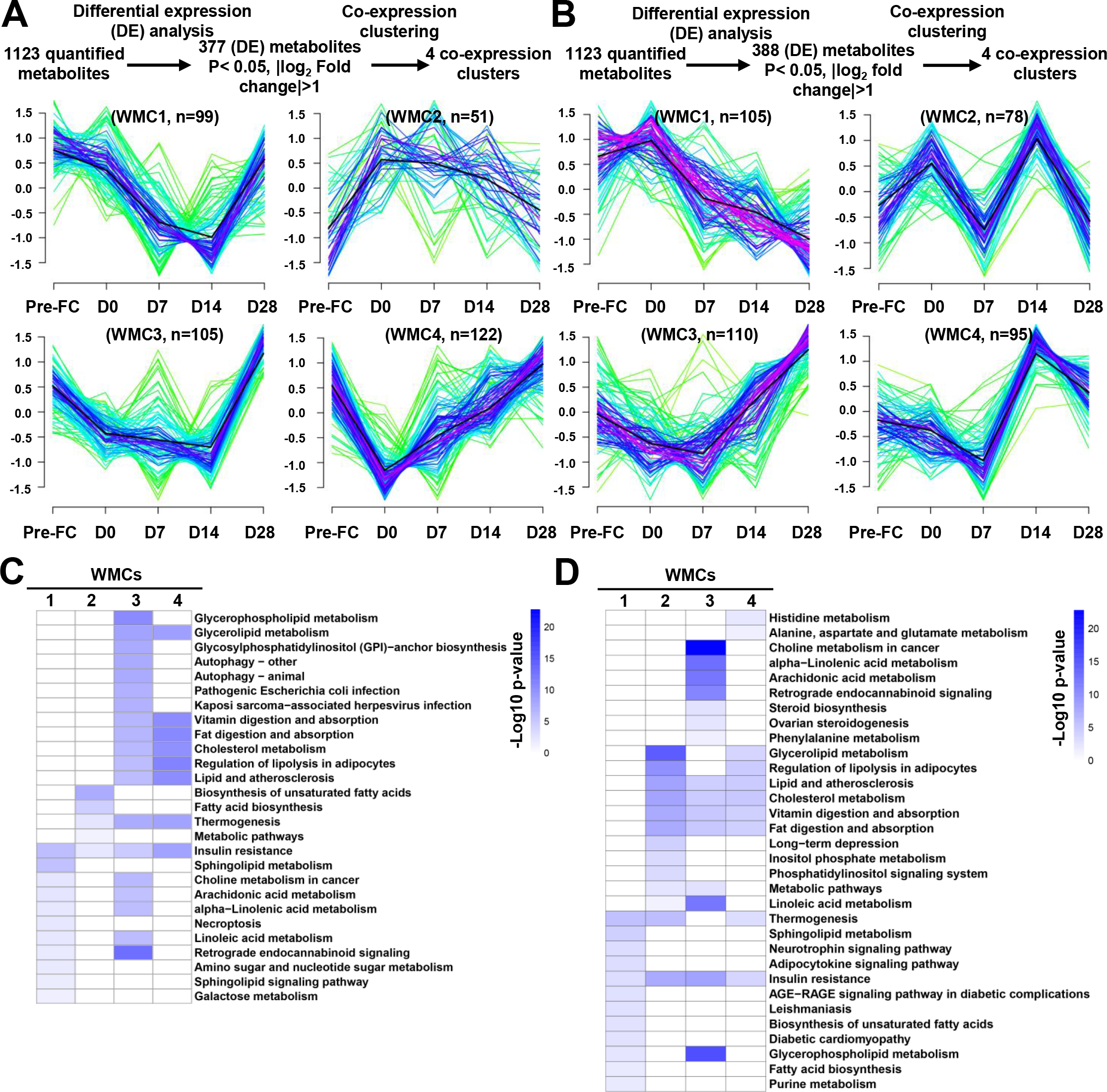


**Figure S11.** **Temporal profiling of** **metabolome during CAR-T cell therapy.** (**A)** Overview of analysis for plasma metabolome in sCR patients. The differentially metabolites (DEMs) were assigned to four whole metabolome clusters (WMCs) on the basis of Mfuzz clustering analysis. Each line indicates the relative abundance of each metabolite and is color-coded by the cluster membership. **(B)** Overview of analysis for plasma metabolome in non-CR patients. The DEMs were assigned to four WMCs according to Mfuzz clustering analysis. Each line indicates the relative abundance of each metabolite and is color-coded by the cluster membership. **(C)** The heatmap displays the functional annotations of WMCs by KEGG in sCR patients. **(D)** The heatmap displays the functional annotations of WMCs by KEGG in non-CR patients.

**Supplementary Tables**

**Table S1.** Demographics and clinical characteristics of patients receiving humanized anti-CD19-CAR-T cell therapy.

**Table S2.** Adverse events within the first month after humanized anti-CD19-CAR-T cell infusion.

**Table S3.** Pathway enrichment analyses of upregulated proteins in B-ALL patients compared with healthy controls at the time point of Day 7.

**Table S4.** Pathway enrichment analyses of upregulated proteins in B-ALL patients compared with healthy controls at the time point of Day 14.

**Table S5.** The enrichment analysis of differentially expressed metabolites in the comparisons of Pre-FC versus HC, Day 0 versus HC, Day 7 versus HC, Day 14 versus HC and Day 28 versus HC groups.

**Table S6.** All differentially expressed metabolites in the comparisons of Pre-FC versus HC, Day 0 versus HC, Day 7 versus HC, Day 14 versus HC and Day 28 versus HC groups.

**Table S7.** Pathway enrichment analyses of upregulated and downregulated proteins in B-ALL patients at the time point of Day 7 compared with pretreatment samples.

**Table S8.** The differentially expressed proteins in the clusters, related to Figure 2A.

**Table S9.** The differentially expressed metabolites in the clusters, related to Figure 2B.

**Table S10.** The functional annotations of differentially expressed metabolites in the clusters by KEGG, related to Figure 2D.

**Table S11**. The differentially expressed metabolites between CS patients and NCS patients at the time point of Day 14.
