# Supplementary material for: Plasma proteomic and metabolomic signatures of B‐ALL patients during CAR‐T cell therapy
Source: Clin Transl Med. 2023 Mar 20;13(3):e1225. doi: 10.1002/ctm2.1225 (PMC10026086; doi:10.1002/ctm2.1225)
Supplement: Supplementary file 2 — Supporting Information [file CTM2-13-e1225-s006.docx]

|  | **Total Patients**  **(n = 20)** | **CS group**  **(n = 13)** | **NCS group**  **(n = 7)** | **Healthy control**  **(n = 22)** |
| --- | --- | --- | --- | --- |
| **Gender (%)**  Female  Male  **Age**  **BMI**  **Prior lines of treatment (≥ 2)**  Vincristine  cyclophosphamide  glucocorticoids | 9 (45.0%)  11 (55.0%)  28.9 ± 12.9  20.4 ± 2.8  18 (90.0%)  20 (100%)  20 (100%)  20 (100%) | 5 (25.0%)  8 (40.0%)  32.8 ± 14.1  21.1 ± 3.1  12 (60.0%)  13 (65.0%)  13 (65.0%)  13 (65.0%) | 4 (20.0%)  3 (15.0%)  21.7 ± 6.4  19.3 ± 2.0  6 (30.0%)  7 (35.0%)  7 (35.0%)  7 (35.0%) | 13 (59.1%)  9 (40.9%)  26.9 ± 2.9  20.5 ± 2.3  - |
| **Disease status** |  |  |  |  |
| Primary refractory | 9 (45.0%) | 5 (25.0%) | 4 (20.0%) | - |
| First relapse | 7(35.0%) | 4 (20.0%) | 3 (15.0%) | - |
| Second relapse | 4 (20.0%) | 4 (20.0%) | 0 (0.0%) | - |
| **Prior transplantation** |  |  |  |  |
| Allogeneic | 3 (15.0%) | 1 (5.0%) | 2 (10.0%) | - |
| Autologous | 1 (5.0%) | 1 (5.0%) | 0 (0.0%) | - |
| **Bone Marrow Blasts** |  |  |  |  |
| < 5% | 5 (25.0%) | 1 (5.0%) | 4 (20.0%) | - |
| ≥ 5%  **Genetics**  BCR-ABL1  MLL-AF4  MEF2D-BCL9  EZH2/KRAS/SETD2 mutation | 15 (75.0%)  7 (35.0%)  1 (5.0%)  1 (5.0%)  1 (5.0%) | 12 (60.0%)  3 (15.0%)  1 (5.0%)  1 (5.0%)  1 (5.0%) | 3 (15.0%)  4 (20.0%)  0 (0.0%)  0 (0.0%)  0 (0.0%) | -  -  -  -  - |

**Table S1.** **Demographics and clinical characteristics of patients receiving** **humanized anti-CD19-CAR-T cell therapy**

Data are presented as mean ± standard deviation (SD) and n/N (%), where N is the total number of patients with available data. CS group represents patients with cytokine storm, whereas NCS group represents patients without cytokine storm.
