# Supplementary material for: Plasma proteomic and metabolomic signatures of B‐ALL patients during CAR‐T cell therapy
Source: Clin Transl Med. 2023 Mar 20;13(3):e1225. doi: 10.1002/ctm2.1225 (PMC10026086; doi:10.1002/ctm2.1225)
Supplement: Supplementary file 3 — Supporting Information [file CTM2-13-e1225-s001.docx]

**Table S2.** **Adverse events within the first month after humanized anti-CD19-CAR-T cell infusion**

|  | **Any** | **Grade 1** | **Grade 2** | **Grade 3** | **Grade 4** |
| --- | --- | --- | --- | --- | --- |
| **CRS**  **CRES**  **General disorders** | 13  2 | 7  0 | 1  0 | 4  2 | 1  0 |
| Fever | 13 | 5 | 5 | 3 | 0 |
| Hypotension | 2 | 0 | 0 | 2 | 0 |
| Hypoxia | 2 | 0 | 0 | 2 | 0 |
| Anemia | 20 | 2 | 8 | 9 | 1 |
| **Neurologic events**  Depressed level of consciousness  Headache  Seizure  **Hematological adverse events**  Lymphocytopenia  Neutrocytopenia  Leukopenia  Thrombocytopenia  APTT prolonged  Fibrinogen decreased  **Chemical laboratory abnormalities**  AST increase  ALT increase  Hypoalbuminemia  Creatinine increase  **Infections**  Viral infection  Bacterial infection  Fungal infection | 2  1  1  20  20  20  15  13  10  6  7  12  0  1  6  3 | 2  0  1  0  0  0  3  13  8  5  6  9  0  0  0  0 | 0  0  0  0  0  0  1  0  0  1  0  1  0  0  0  0 | 0  1  0  0  2  1  4  0  2  0  1  2  0  1  6  3 | 0  0  0  20  18  19  7  0  0  0  0  0  0  0  0  0 |

Data are presented as mean ± standard deviation (SD) and n/N (%), where N is the total number of patients with available data. Individual symptoms of AEs were graded using CTCAE, version 4.03.
